# Exploring electrode montasges to optimize the non-invasive clinical recording of auditory evoked AP/wave I

**DOI:** 10.1016/j.cnp.2026.07.001

**Published:** 2026-07-04

**Authors:** Ludivine Beaud-Henry, Aurélien Mulliez, Alann Renault, Katia Bouton, Paul Avan, Fabrice Giraudet

**Affiliations:** a UMR 1107 INSERM, Neurosensory Biophysics Team, Clermont-Ferrand, France; b Service de médecine interne, centre de référence pour les maladies auto-immunes et auto-inflammatoires systémiques rares d'Auvergne, CHU Gabriel-Montpied, Clermont-Ferrand, France; c Direction de la Recherche Clinique et de l'Innovation, CHU de Clermont-Ferrand, France; d Laboratoire d'Acoustique de l'Université du Mans LAUM -. UMR CNRS 6613, Le Mans, France; e Université Paris Cité, Institut Pasteur, AP-HP, INSERM, CNRS, Fondation Pour l'Audition, Institut de l'Audition, IHU reConnect, F-75012 Paris, France; f Department of Medical Genetics, CHU de Clermont-Ferrand, France

**Keywords:** Electrocochleography, Auditory brainstem response, Electrode montage, AP/wave I amplitude, Tiptrode electrode, Cochlear synaptopathy, Endocochlear hydrops, Auditory neuropathy

## Abstract

**Objectives:**

In clinical routine, the use of electrocochleography (Ecog) has sparked renewed interest with new less invasive electrodes (gold eartip electrode) to document complex pathological entities such as endocochlear hydrops, auditory neuropathies, cochlear synaptopathy but also monitoring intracranial pressure changes. To minimize variability due to methodological factors, this study aims to compare, in normal-hearing adults (both gender), the compound action potential (AP)/wave I amplitude recorded with earlobe and ear canal electrodes as well as the effect of click polarity (rarefaction vs. condensation).

**Results:**

All electrophysiological tests were collected using a conventional clinical evoked potential measuring system. The results for AP/wave I latency remained relatively stable between the Auditory Brainstem Response (ABR) and Ecog recording protocols, with a trend toward shorter latencies with rarefaction polarity in women. Our findings support the use of the ear canal electrode with both ABR and Ecog protocols; a vertical electrode montage and rarefaction click polarity consistently provide significantly higher amplitudes for AP/wave I in both genders.

**Discussion:**

While Ecog has long been used for the objective diagnosis and monitoring of endolymphatic hydrops, Ecog can be also used in the diagnosis of auditory neuropathy. Recent findings from animal studies suggest that the emergence of AP/wave I is a sensitive indicator of age-related or noise-induced cochlear synaptic loss (cochlear synaptopathy). It therefore seems essential to have an optimized Ecog protocol for a more sensitive evaluation of the morphology of AP/wave I as indispensable tool to improve understanding of different pathological entities.

## Highlights

• Ear-canal electrodes with a vertical montage provide the highest AP/wave I amplitudes.
• Rarefaction polarity consistently improves AP/wave I morphology.
• Improved AP/wave I capture strengthens Ecog utility for diverse auditory pathologies.

## 1 Introduction

To understand the functional status of the brain, neuro-electric signals, called electroencephalogram (EEG), can be measured through electrodes placed on the scalp. The international 10–20 system is a method to standardize the placement of electrodes in EEG, based on anatomical landmarks of the skull (Klem et al., 1999). Evoked potentials (auditory, visual and somatosensory) are micro-voltage responses buried in the EEG in response to repetitive external stimuli (auditory, visual or electrical), generated within a localized part of the neural pathways. Evoked potentials are an essential non-invasive tool for assessing the function and integrity of the nervous system and for diagnosing disorders (Guérit et al., 2002).

Evoked potentials, measured using surface skin electrodes placed on the scalp, are far-field recordings. The alignment of the electrode montage with the equivalent dipole source significantly influences the morphology of the waveforms. Whenever possible, the electrode impedance should be as low as possible and inter-electrode impedance should be relatively similar across electrodes to improve the quality of recordings (Durrant et al., 2022; Hall, 2007).

The use of cup electrodes requires a gentle scalp scarification with an skin preparative gel (risk of scalp compromise in newborns and infants), and a conduct paste prior electrode placement. Cup electrodes provide an attractive option for neonatal electrophysiology due to cephalic characteristics of newborns, such as the small head. For auditory evoked potentials recording, this method has gradually given way to the use of sterile, disposable, adhesive electrodes which has been followed by a shift in the position of the electrodes from vertex (Cz) to high forehead (Fz) and from the earlobes to the mastoids (Giraudet et al., 2026).

These electrodes montages have now become « traditional » in audiology routine practice (Durrant et al., 2022; Hall, 2007). However, the surface skin still needs to be prepared, but the application of these commercial electrodes does not require additional conductive paste. Unfortunately, the single-use gel-coated electrodes show some limitations, such as the drying of the conductive gel, the expiration date and the painful discomfort removing of the electrodes in pediatric population.

Furthermore, the recent development of new; less invasive electrodes (e.g., the gold-coated foam electrode Tiptrode) has sparked renewed interest in clinical applications of electrocochleography (ECochG) (Gibson, 2017; Simpson et al., 2020). The use of ECochG for assessment of cochlear and auditory-nerve function remains indispensable for monitoring endocochlear hydrops, auditory neuropathies, cochlear synaptopathy (Giraudet et al., 2026).

To overcome diagnostic difficulties, it is essential to determine the most suitable electrophysiological methodology for providing an objective, non-invasive assessment offering enhanced identification of the components of the ECochG (cochlear microphonic, summation potential, action potential (AP)/wave I) (Gibson, 2017; Giraudet et al., 2026).

To reduce variability due to methodological factors and to determine the optimal placement of the electrodes, this study aims to compare, in normal-hearing adults of both genders, the AP/wave I amplitude using earlobe and ear canal electrodes, based on a clinical evoked potential measuring device. The effect of click polarity (rarefaction vs. condensation) on AP/wave I amplitude is also analyzed.

## 2 Methods

### 2.1 Subjects and study design

This study received approval from the national ethics committee (IDRCB approval number: 2018-A02525–50; clinical trial registration number NCT04198909). Written informed consent was obtained from each participant before starting the experiment. The study included 47 young adults (24 women) aged between 18 and 25 years.

Tests were conducted in an acoustically controlled and soundproofed room. Prior to evaluation, an otoscopic examination was performed to ensure that the ear canal was free of wax and that the tympanic membrane appeared normal. All participants underwent an audiologic evaluation, including tympanometry, middle ear muscle reflex (MEMR) and air-conduction thresholds.

Inclusion criteria were a good general health, a normal tympanogram (type A), normal-hearing thresholds (≤ 20 dB HL) based on pure-tone audiometry at octave frequencies between 0.25 and 8 kHz, and the presence of ipsilateral MEMRs at 0.5, 1, 2 and 4 kHz. Exclusion criteria included absence of informed consent, a history of otologic disorders, the presence of earwax, middle-ear pathology, sensorineural hearing loss, hearing complaints (such as tinnitus or speech-intelligibility difficulties), any (known) disorders (e.g., neurological, psychiatric, metabolic, or cardiovascular).

### 2.2 Pure-tone audiometry

The monaural air-conduction threshold was measured in both ears for each participant at 0.125, 0.25, 0.5, 0.75, 1, 1.5, 2, 3, 4, 6, 8, 9, 10, 11.2, 12.5, and 14 kHz using a pure-tone audiometer (HDA 300 circumaural headphones, Sennheiser and Equinox, Interacoustics software ver. 2.11.0). Procedures and requirements for pure-tone air-conduction threshold audiometry complied with ISO 8253–1:2010 standard., with 5 dB intensity increments following a modified Hughson-Westlake procedure. The 4-frequency pure-tone average (PTA) was defined as the average of hearing thresholds at the frequencies 0.5, 1, 2 and 4 kHz (according to the 02/1 bis recommendations of the Bureau International d'AudioPhonologie).

### 2.3 Tympanometry - Middle ear muscle reflex

Conventional tympanometry was performed in both ears using a diagnostic middle-ear analyzer (Titan, Interacoustics, software ver.3.7.2) according to ISO 60645-5:2005 standard. The device uses a probe tone frequency of 226 Hz and a pressure-sweep range of +300 to −300 daPa, with a selected automatic speed of 400 ± 40 daPa/s. Tympanometry was considered normal (type A) when the peak pressure fell between −100 and + 100 daPa with a compliance between 0.3 and 1.3 ml. Middle ear muscle reflexes (MEMR) were measured, according to ISO 60645-5:2005 standard, in both ears in response to ipsilateral elicitor pulsed pure tones at 0.5, 1, 2 and 4 kHz, presented at increasing sound levels from 50 to 100 dB HL in 5 dB steps. Threshold at each elicitor frequency was defined as the lowest stimulus level producing a reduction in compliance of 0.02 ml or greater, observed twice. As with the PTA, the MEMR threshold was defined as the average threshold obtained across the four elicitor frequencies for each ear in both genders.

### 2.4 Electrophysiological tests

All electrophysiological tests were performed using two-channel auditory evoked-potentials measuring system (Eclipse EP-25, Interacoustics, software ver.4.6.0.33), under standard conditions in a sound-attenuated chamber, with room temperature 4.6.0.33) under standard conditions in a sound‑attenuated chamber, with room temperature controlled and lighting dimmed. Participants were comfortably positioned in a supine posture on an examination bed and instructed to remain as still and relaxed as much as with their eyes closed. For all subjects the right ear was tested.

**Fig. 1:**
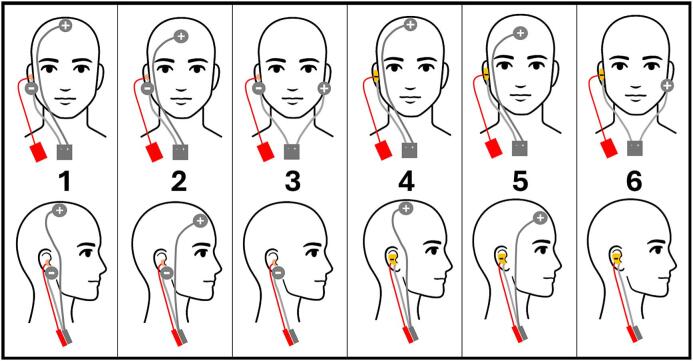
Schematic representation of the different electrode montages. The sketches illustrate the position of the passive (−) and active (+) electrode resulting in six different configurations: montage 1 = earlobe/Cz, montage 2 = earlobe/Fz, montage 3 = earlobe/earlobe, montage 4 = ear canal/Cz, montage 5 = ear canal/Fz, montage 6 = ear canal/ear canal. Surface electrodes were cupule electrodes (Cz, Fz, earlobe, in grey). Tiptrode electrode (in yellow) was placed in the ear canal. Acoustic stimulation was delivered via an insert earphone with a tube extended (in red) by a single-use foam tip (montage 1, 2, 3) or a Tiptrode (tip covered a thin gold foil). Grey box: pre-amplifier, ground electrode (positioned laterally at the forehead) omitted. (For interpretation of the references to color in this figure legend, the reader is referred to the web version of this article.)

The skin of the electrode sites (Cz, Fz, earlobe, first ear canal bend, forehead) were prepared using an abrasive paste (NuPrep skin prep Gel, Weaver and Company). The electrode impedance was less than 3 kΩ, with no inter-electrode impedance exceeding 1 kΩ. The recording electrodes were Ag/AgCl surface cupules and were positioned using skin conduction paste (Elefix EEG Paste, Nihon Kohden). The electrode is secured with a small gauze pad. For all montages, the common ground electrode was placed laterally on the left side of the forehead (Fp1). The non-inverting active electrode (tested right ear) was either a cupule electrode placed on the earlobe or a gold-foil covered foam tip inserted in the ear canal (Tiptrode, Etymotic Research, Elk Grove Village, IL, USA). The inverting passive electrode was either a cupule electrode placed on the vertex (Cz) or on the high center forehead (Fz) or in the ear canal (Tiptrode). Fig. 1 illustrates the different electrode montages. In order to ensure consistent insertion depth, the foam tips and the Tiptrode were inserted safely and completely with their outer edges flush to the entrance of the external auditory meatus.

Rarefaction and condensation polarity click stimuli (100 μs duration, presentation rate 17.7/s) were delivered separately and monoaurally at fixed 70 dB nHL (95 dB peak SPL) via insert earphones (ER-3a - Etymotic Research, Inc., Elk Grove Village, IL). The non-test left ear remained unobstructed. Data were collected using two consecutive different “manufacturer” recording protocols. For the ABR protocol, signals were band-pass filtered between 33 and 1500 Hz (with 6 dB/octave filter slope). For the ECochG protocol the settings was a 10–5000 Hz passband filter (with 6 dB/octave filter slope). No notch filter (50–60 Hz) was applied.

The effects of the six different electrode montages on electrophysiological measurements were evaluated using both ABR and ECochG protocols. For each recording protocol, polarity and electrode montage, 1000 sweeps were collected with a minimum of two repeatable averaged waveforms (a total of 24 electrophysiological waveforms per participant). The order of stimulus polarities and montage recordings was randomized. The epoch time was set at 21 ms for the ABR protocol and 10 ms for the ECochG protocol, including a 3 ms prior to click presentation, thus setting a baseline (Fig. 6).

Prior to data analysis, two collected waveforms (each comprising 1000 sweeps) were averaged into a single grand average. For each resulting waveform, two experts conducted independent reviews and annotated baseline, wave I for ABR protocol and AP for ECochG protocol. The AP/wave I amplitude was determined as the difference between the baseline and the AP/wave I peak (baseline-to-peak amplitude). The baseline was marked at the onset of wave I (at the bottom of the shoulder, around 1 ms) or at the onset of the SP (at the onset of the shoulder, around 0.5 ms).

### 2.5 Statistics

Statistics and figures were performed using Sigmaplot (version 11.0, Systat Software) and Stata software (version 15, StataCorp, College Station, US). Descriptive statistics are shown as numbers and percentage for categorical data and as mean ± standard deviation (SD) and range (minimum – maximum) for continuous data. Comparison of continuous data between sex were performed using Student's *t*-test (when data distribution is normal) or using Mann & Whitney's test (when data not normal). Normality was assessed graphically and using Shapiro Wilk's test. Tests were two-sided and a *p*-value <5% was considered statistically significant.

## 3 Results

### 3.1 Pure-tone audiometry - Middle ear muscle reflex

**Fig. 2:**
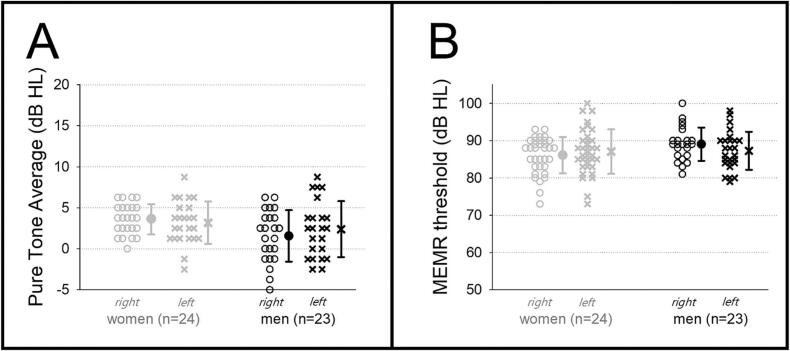
Participants audiological characteristics. Panel A: Distribution of individual and mean ± SD pure-tone audiometric thresholds (PTA in dB HL) for the right (circles) and the left (crosses) ears in women (grey symbols, *n* = 24) and men (black symbols, *n* = 23). Panel B: Distribution of individual and mean ± SD middle ear muscle reflex (MEMR) threshold (in dB HL, mean threshold across 0.5, 1, 2, and 4 kHz) for the right (circles) and the left (crosses) ears in women (grey symbols, n = 24) and men (black symbols, n = 23).

The entire study group comprised 47 subjects, including 24 women (51%). The mean age was 20.8 ± 1.8 years for men and 21.4 ± 1.9 years for women. All participants had normal audiometric thresholds (≤ 20 dB HL) in both ears. Individual PTAs for the right and left ears, by gender, are shown in Fig. 2a. The PTAs ranged from −5 ± 2 to 9 ± 3 dB HL; mean PTA was 2 ± 2 dB HL for men and 3 ± 3 dB HL for women. There was no statistically significant difference in PTA between gender and ear side.

Peripheral auditory pathways were further investigated by measuring middle ear muscle reflex (MEMR), one of the major efferent descending systems. Fig. 2B illustrates the distribution of MEMR thresholds, average threshold obtained across the four elicitor frequencies, for the right and left ear in both genders. The MEMR thresholds ranged from 73 to 100 dB HL, with a mean 88 ± 5 dB HL for men and 87 ± 5 dB HL for women. There was no statistically significant difference between genders or between ear sides.

### 3.2 Electrophysiological tests

**Fig. 3:**
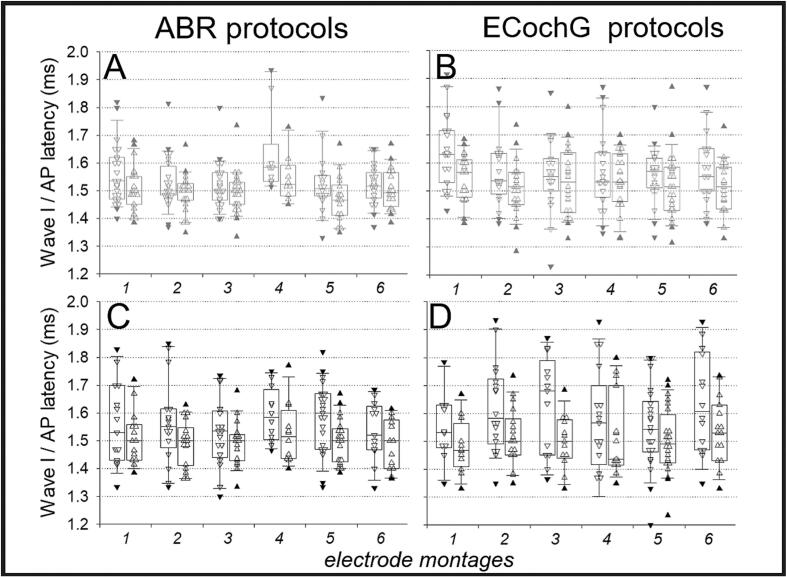
Influence of electrodes montage and click polarity on AP/ wave I latency in both genders. Boxplot of AP/wave I latency according protocol (ABR panel A-C, ECochG panel B—D), electrode montage, gender group (women in grey symbols, panel A-B, n = 24; men in black symbols, panel C—D, n = 23) and click polarity (condensation in downward-open triangles, rarefaction in upward-open triangles). Electrode montages: montage 1 = earlobe/Cz, montage 2 = earlobe/Fz, montage 3 = earlobe/earlobe, montage 4 = ear canal/Cz, montage 5 = ear canal/Fz, montage 6 = ear canal/ear canal.

**Fig. 4:**
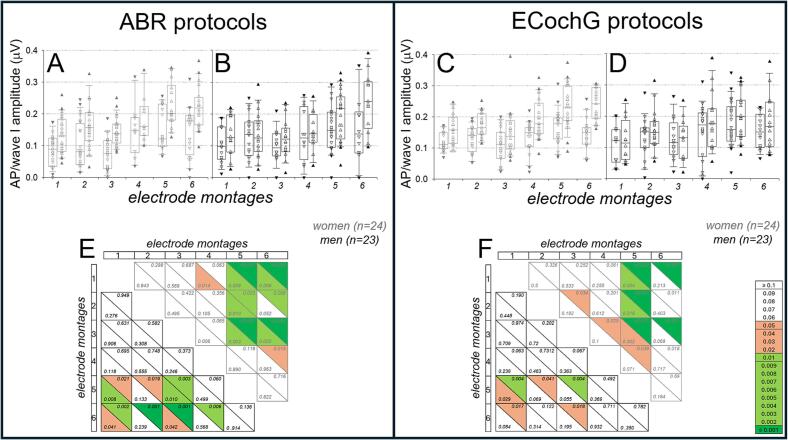
Influence of electrodes montage, recording protocol and click polarity on AP/ wave I amplitude in both genders. Boxplot of AP/wave I amplitude according to protocols (ABR panel A&B, ECochG panel C&D), electrode montage, gender (women in grey symbols, panel A&C, n = 24; men in black symbols, panel B&D, n = 23) and click polarity (condensation in downward-open triangles, rarefaction in upward-open triangles). Electrode montages: montage 1 = earlobe/Cz, montage 2 = earlobe/Fz, montage 3 = earlobe/earlobe, montage 4 = ear canal/Cz, montage 5 = ear canal/Fz, montage 6 = ear canal/ear canal. In panels E and F, the matrix presents the detailed intra-group results of the statistical comparison of the AP/wave I amplitude across the different recording configurations (montage, click polarity) women (in grey) and in men (in black) and according to ABR or ECochG protocol. The color scale reflects the magnitude of the statistical significance (*p*-values).

Fig. 3 represents the individual values and boxplots for AP/wave I latency for ABR and ECochG protocols, electrode montages, gender and click polarity. Mean AP/wave I latency values obtained for the different electrode montages (1 to 6) range from 1.50 ± 0.08 ms to 1.54 ± 0.10 ms in women and from 1.49 ± 0.09 ms to 1.52 ± 0.13 ms in men with rarefaction polarity. With condensation polarity, across the six different electrode montages, AP/wave I latency range from 1.54 ± 0.10 ms to 1.60 ± 0.13 ms in women and from 1.56 ms ± 0.13 ms and 1.59 ms ± 0.16 ms in men. There was no statistically significant difference in AP/wave I latency between the two groups in terms of protocol, electrode montages, gender and click polarity. For each gender, regardless of the recording conditions (protocol, electrode montage, click polarity) the latency for AP/wave I remained relatively stable. However, Fig. 3 highlights four key observations. For the majority of the measures, the variability of the distribution of latencies was greater with condensation polarity. In many boxplots of rarefaction polarity, the centered median reflects a normal distribution of latency values. Moreover, the median of the boxplots for rarefaction polarity is often lower than for those for condensation polarity. Although statistical analyses reveal no significant difference, there is a slight tendency for women to show shorter AP/wave I latencies. (See Fig. 4).

Fig. 4 represents the individual values and boxplots for AP/wave I amplitude for ABR and ECochG protocols, electrode montages, gender and the click polarity.

Amplitude mean values of wave I in women with the ABR recording protocol ranged from 0.14 ± 0.06 μV to 0.23 μV ± 0.07 μV with rarefaction polarity and from 0.07 ± 0.05 μV to 0.15 μV ± 0.08 μV with condensation polarity across the six different electrode montages. For the ECochG protocol and electrode montages 1, 2 and 3, the AP amplitude ranged from 0.15 ± 0.07 μV to 0.17 ± 0.04 μV with rarefaction polarity and from 0.11 ± 0.06 μV to 0.013 ± 0.04 μV with condensation polarity. For electrode montages 4, 5 and 6 with ECochG protocol, AP amplitudes ranged from 0.19 ± 0.06 μV to 0.24 ± 0.07 μV with rarefaction polarity and from 0.14 ± 0.05 μV to 0.17 μV ± 0.06 μV with in condensation polarity in women.

For men, the amplitude of wave I recorded with the ABR protocol ranged from 0.12 ± 0.06 μV to 0.24 ± 0.09 μV with rarefaction polarity and from 0.10 ± 0.06 μV to 0.16 μV ± 0.08 μV with condensation polarity across the six different electrode montages. For ECochG protocol and electrode montages 1, 2 and 3, the AP amplitude ranged from 0.12 ± 0.07 μV to 0.16 ± 0.06 μV with rarefaction polarity and from 0.11 ± 0.06 μV to 0.13 ± 0.07 μV with in condensation polarity. For electrode montages 4, 5 and 6 with ECochG protocol, AP amplitudes ranged from from 0.18 ± 0.09 μV and 0.20 ± 0.08 μV with rarefaction polarity and from 0.15 ± 0.09 μV to 0.18 ± 0.07 μV with condensation polarity in men.

Statistical analyses revealed no significant sex-related differences in AP/wave I amplitudes. The intra-group statistical comparison analysis (statistical significance, *p*-values) of the AP/wave I amplitude across the different recording modalities (montage, polarity, gender, recording protocol) is presented in detail in the matrix (Fig. 4 panel E & F).

**Fig. 5:**
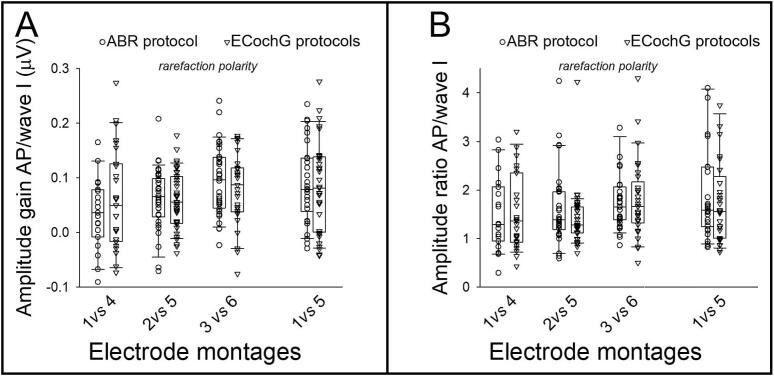
The AP/wave I amplitude enhancement according electrode montages. The graphs in panels A and B illustrate the increase in AP/wave I amplitude (in absolute values (μV) and as a ratio) obtained by comparing similar electrode montages but with different active electrode (ear canal versus earlobe electrode) according to ABR or ECochG protocols.

Fig. 5 (panels A and B) illustrate the enhancement of amplitude of AP/wave I obtained using the ear canal electrode compared to the earlobe electrode. This increase in amplitude can reach an average ratio AP/Wave I of 1.40 ± 0.55 to 2.14 ± 1.9. With the ear canal electrode, there is no significant difference in amplitude enhancement between the electrode montage 5 (ear canal/Fz) and the electrode montage 6 (ear canal/ear canal). On average, the amplitude increased by 0.089 ± 0.071 μV when comparing the conventional electrode montage for recording the ABR (montage 1) to the recommended ECochG montage with an ear canal electrode (montage 5), corresponding to a ratio of 2.12 ± 1.62 (with both ABR and ECochG protocols).

**Fig. 6:**
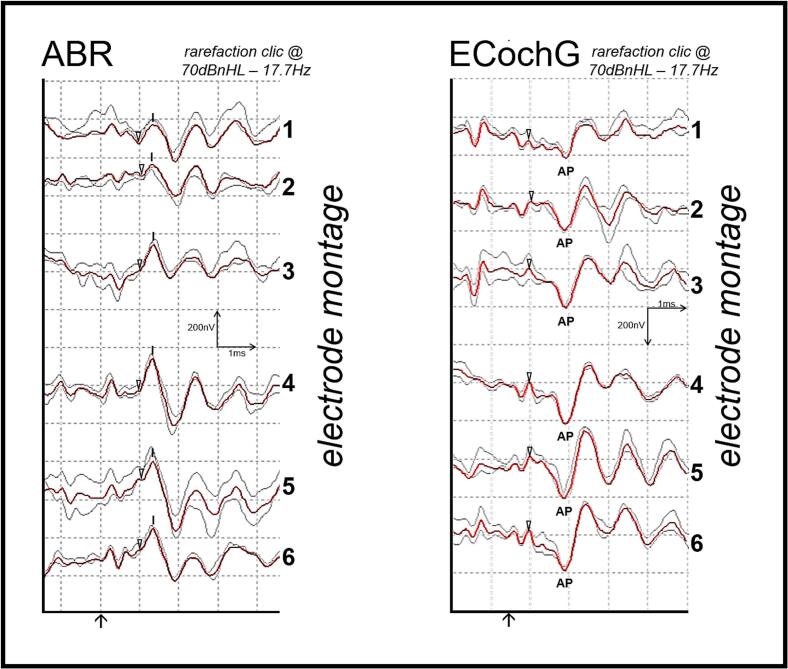
Grand-average of ABR and ECochG waveforms. Two recorded waveforms (1000 sweeps, in grey) evoked by a 70dBnHL rarefaction click were superimposed and averaged to obtain a grand-averaged response (in red) for each montage in both the ABR and ECochG protocols. Wave I of the ABR is labeled with the Roman numeral “I,” action potential of the ECochG is labeled “AP”, upward arrow indicates the time scale origin, downward open triangle indicates the baseline marked at the onset of the wave I (bottow of the shoulder, at about 1 ms) or at the onset of the SP (onset of the shoulder, at about 0.5 ms). (For interpretation of the references to color in this figure legend, the reader is referred to the web version of this article.)

## 4 Discussion

While electrocochleography (ECochG) was once very popular, its invasive characteristics and the emergence of new imaging techniques have gradually led to a decline in interest and to be gradually set aside. However, in recent years, new and less invasive electrodes (placed in the ear canal, also called Tiptrode) have become relevant for obtaining high-quality recordings and enabling a more refined evaluation of complex pathological entities such as auditory neuropathies (De Siati et al., 2020) and cochlear synaptopathy (Vasilkov et al., 2023). ECochG provides a conventional useful and reliable diagnostic tool for endolymphatic hydrops (Gerenton et al., 2015; Deng et al., 2026) and for intraoperative monitoring (Casile et al., 2024; Giraudet et al., 2017; Lourenço et al., 2018).

Our study aimed to optimize ECochG procedures by examining various experimental conditions, such as acoustic stimulus characteristics and electrode placement. Adopting a pragmatic approach that can be directly applied in clinical practice with conventional auditory evoked-potential systems, we focused our analyses on evaluating the latency and amplitude of AP/wave I in both women and men with normal hearing. In addition to the stimulation parameters (click polarity) and recording parameters (electrode type and placement), we examined the electrophysiological waveforms obtained using two “manufacturer” protocols: Auditory Brainstem Responses (ABR) and ECochG.

In literature and in clinical practice, many different methods have been used to calculate AP/wave I amplitude, such as the difference in amplitude between the prestimulus baseline (the time period before stimulus presentation) and the AP/wave I peak, or between the AP/wave I peak and the subsequent trough (Durrant et al., 2022; Ferraro, 2010; Hall, 2007; Wuyts et al., 1997). Because the trough is often easier to identify, many authors prefer to measure the AP/wave I amplitude using this peak-to-trough approach (Atcherson et al., 2012, Bramhall, 2021, McFarlane and McFarlane and Sanchez, 2023). Nevertheless, for all the data collected and for subsequent analysis, we opted for the conventional approach employed in ECochG of determining the AP/wave I amplitude as the difference between the baseline and the AP/wave I peak (baseline-to-peak amplitude) (Liberman et al., 2016). Many studies recommend using this procedure to assess the SP/AP ratio. Indeed, an elevated SP relative to AP is a diagnostic indicator of endolymphatic hydrops/Meniere's disease (Ferraro, 2010; Wuyts et al., 1997; Deng et al., 2026).

As expected, the results for AP/wave I latency remained relatively stable between the ABR and ECochG protocols. Despite a tendency toward shorter latencies with rarefaction polarity in women (consistent with previous study, Michalewski et al., 1980), there were no significant differences in AP/wave I latency between polarity, electrode montage or gender. In routine clinical practice, the focus of auditory evoked potentials (ABR and ECochG) is on examining waveform latencies, as these are largely insensitive to various electrode montages (Durrant et al., 2022; Hall, 2007).

Our study focused on optimizing AP/wave I amplitude through electrode placement. First, our findings show that, regardless of gender or electrode montage, rarefaction polarity provides a more consistently stronger AP/wave I amplitude than condensation polarity. As in previous studies (Bauch and Olsen, 1990; Ferraro, 2010; Prendergast et al., 2018), we consistently obtained larger AP/wave I amplitude recordings using the ear canal electrode (Tiptrode) compared to earlobe electrodes, in both the ABR and ECochG protocols. While the positioning of the vertex (Cz), high forehead (Fz) and earlobe electrode can be easily reproducible between different operators, the positioning and, more particularly, the depth of insertion of the ear canal electrode may be inconsistent. The outer edge of the ear canal meatus was chosen as a visual landmark to ensure consistent depth of full insertion of the ear canal electrode across participants (as for the foam tips).

Our findings show that the amplitude of AP/wave I was similar in montage 5 (vertical) and montage 6 (horizontal) recordings using an ear canal electrode. In their recent report, McFarlane and Sanchez observed a larger AP/Wave I amplitude in the vertical montage in the large majority of their volunteers (McFarlane and Sanchez, 2023). Using a horizontal montage (Tiptrode in both ear canals) Vasilkov and colleagues described higher trough-to-peak AP amplitude after high-pass filtering than conventional measurement (Vasilkov et al., 2023). However, this filtering requires post-processing using an additional processing module, which is less user-friendly in routine clinical practice.

Animal studies indicated that AP/wave I amplitude can be used as an electrophysiological marker of age-related or noise-induced cochlear synaptic loss (Kujawa and Liberman, 2009; Sergeyenko et al., 2013). Several studies in humans have shown reductions in AP/wave I amplitude associated with self-reported tinnitus (Bramhall et al., 2019; Schaette and McAlpine, 2011). Others recent studies have also reported that SP/AP ratio emerged as a sensitive indicator of cochlear synaptic loss (cochlear synaptopathy) in humans (Bramhall, 2021; Liberman et al., 2016; Mehraei et al., 2016; Vasilkov et al., 2023; Verhulst et al., 2016).

In conclusion, this study presents a pragmatic approach for obtaining high-quality ECochG measurement using a conventional auditory evoked system, with a standard electrophysiological acquisition software (rather than dedicated ECochG software) and the ear canal electrode (Tiptrode). To improve participant comfort and benefit future patients, we recommend using ear canal electrodes instead of peri- or trans-tympanic needle electrodes even though the latter record stronger electrophysiological responses. The recommended recording parameters -namely a vertical montage (Fpz–ear canal, montage 5) and rarefaction click polarity- consistently produce a significantly higher amplitude for AP/wave I in both genders. Thus, the evaluation of AP/wave I morphology (latency and amplitude) seem to be one of an indispensable tool to improve understanding of several pathological entities such as auditory neuropathies, cochlear synaptopathy, endocochlear hydrops.

## Declaration of competing interest

The authors declare that they have no known competing financial interests or personal relationships that could have appeared to influence the work reported in this paper.
